# Effect of cognitive behavioral therapy-based counseling on perceived stress in pregnant women with history of primary infertility: a controlled randomized clinical trial

**DOI:** 10.1186/s12888-021-03283-2

**Published:** 2021-05-31

**Authors:** Farideh Golshani, Shirin Hasanpour, Mojgan Mirghafourvand, Khalil Esmaeilpour

**Affiliations:** 1 Student Research Committee Department of Midwifery, Nursing and Midwifery Faculty, Tabriz University of Medical Sciences, Tabriz, Iran; 2 Women’s Reproductive Health Research Center, Tabriz University of Medical Sciences, Tabriz, Iran; 3 Midwifery Department, Social Determinants of Health Research Center, Tabriz University of Medical Sciences, Tabriz, Iran; 4 Faculty of Education and Psychology, Tabriz University, Tabriz, Iran

**Keywords:** Cognitive behavioral counseling, Perceived stress, Depressions, Anxiety, Quality of life, Primary infertility

## Abstract

**Background:**

Given the prevalence of infertility and consequences of stress, anxiety, and depression during pregnancy and after childbirth, this study aimed to determine the effect of group cognitive behavioral therapy (CBT)-based counseling on perceived stress (primary outcome), anxiety, depression, and quality of life (QoL) of pregnant women with a history of primary infertility.

**Method:**

This controlled randomized clinical trial was conducted on 56 pregnant women with a history of primary infertility referred to Infertility Clinic of Al-Zahra Teaching Hospital of Tabriz. The participants were divided into the intervention (*n* = 28) and control (n = 28) groups using block randomization. The intervention group received group CBT-based counseling after the 14th week of the pregnancy: six in-person sessions and two telephone sessions once per week. The control group received routine care. The Perceived Stress Scale (PSS), Edinburgh Postnatal Depression Scale (EPDS), Van den Bergh’s Pregnancy-Related Anxiety Questionnaire (PRAQ), and Quality of Life in Pregnancy (Gravidarum) (QOL-GRAV) were completed through interviews before and 4 weeks after the intervention by the researcher.

**Results:**

There was not any between-group difference in socio-demographic characteristics, except the gestational age and husband educational level (*p* > 0.05). Both of these variables were adjusted in ANCOVA. After the intervention, the mean scores of perceived stress (mean difference: − 7.3; confidence interval: 95%, from − 0.9 to − 5.6; *p* < 0.001) and anxiety (mean difference:-14.7; confidence interval: 95%. from − 20.6 to - 8.8; p < 0.001) were significantly lower in the intervention group. The mean depression score in the intervention group was lower than the control; however, this between-group difference was not significant (mean difference: − 1.95; confidence interval: 95% from − 3.9 to 0.2; *p* = 0.052). The mean score of quality of life in pregnancy was significantly higher in the intervention group than the control (mean difference: − 5.4; confidence interval: 95% from 3.4 to 7.4; *p* < 0.001).

**Conclusion:**

CBT counseling can affect the perceived stress, anxiety, and quality of life of pregnant women with a history of primary infertility. As a result, this counseling approach is recommended along with other counseling approaches to improve the mental health of pregnant women with a history of infertility.

**Trial registration:**

IRCT Registration Number: IRCT20111219008459N12, registered on 10/11/ 2018.

## Background

Infertility is defined as the inability to become pregnant after one year of unprotected coitus [1]. Acording to studies, about 11 to 51 million people in the world suffer from some form of infertility; one in six couples of childbearing age worldwide has this problem [2]. As shown in the literature, the mean rate of infertility in the world is 10% [3]. Female infertility occurs in about 37% of all infertile couples [4]. It ranged from 0.6 to 3.4% for the primary infertility and 8.7 to 32.6% for the secondary infertility [5]. In a review study, the prevalence of infertility was reported 17.3% in Iran [3]. The number of infertility treatments is growing and these treatments are stressful for infertile women. The associated psychological pressures and negative behavioral states can threaten the in vitro fertilization (IVF)/intra cytoplasm sperm injection (ICSI) outcomes [6].

Stress is defined as an organism’s total response to environmental demands or pressures which is perceived as a threat to their abilities and resources and endangers their health [7]. Stress during pregnancy is related to neonatal delayed motor development, and cognitive and behavioral disorders. Moreover, it can cause such complications as pregnancy poisoning and spontaneous abortion [8]. Pregnancy-induced anxiety is a strong factor in the prediction of negative outcomes, such as poor fetal development, preterm delivery, low birth weight, and impaired psychomotor development [9]. Depression is the most common mental disorder during pregnancy period [10]. Untreated maternal depression during pregnancy causes growth disorder, low birth weight, preterm delivery, attention deficit hyperactivity disorder, and fetal arrhythmia [11]. Investigations on the quality of life of couples undergoing IVF treatment showed that it was lower in women than in men [12].

Different studies have recommended several psychological interventions, including Cognitive Behavioral Therapy (CBT) to improve depression and anxiety [13, 14]. CBT helps patients to understand their distorted thinking patterns and dysfunctional behaviors. The basis of CBT is to change the cognitive process. According to this theory, the experience of behavior alone is not sufficient; rather it is an individual’s interpretation of that experience that causes a psychological disorder. Such methods, known as CBT, are used to moderate misunderstanding and misinterpretation of important circumstances of life [15].

LoGiudice et al. (2018) performed a systematic review to investigate the effect of complementary therapies on psychological factors in women undergoing IVF and observed their effectiveness in reducing anxiety, depression, and stress, and improving quality of life during pregnancy [16]. Klerk et al. (2005) investigated the effectiveness of psychological counseling on women going through IVF and found no significant between-group difference [17]. Hämmerli et al. (2009) failed to show the effectiveness of psychological interventions in improving mental health (depression and anxiety) [18]. In LoGiudice review study, the effect of all complementary medicine treatments such as yoga, mind-body techniques and cognitive behavioral therapy on the psychological problems of infertile women undergoing or about to be undergoing IVF cycle, was reported; while the next two studies only measured the effect of psychological counseling in these women. None of the above studies have examined the effect of psychological counseling on the mental health of pregnant women following assisted reproduction technologies (ART).

Taking into account the anxiety and stress in pregnant women with a history of infertility [19], their adverse effects [20], and contradiction in the literature results, the present study was conducted to determine the effectiveness of group cognitive behavioral therapy-based counseling on perceived stress (primary outcome), anxiety, depression and quality of life in pregnant women with history of primary infertility. The present study developed following hypothesis: CBT counseling reduces perceived stress, anxiety, and depression of pregnant women with a history of primary infertility and improves their quality of life.

## Method

### Research design and participants

This controlled randomized clinical trial was conducted on 56 pregnant women aged between 20 and 40 years old with a history of primary infertility referred to Infertility Clinic of Al-Zahra in Tabriz, Iran. Sampling was conducted from November 2018 to June 2019. Inclusion criteria were age range between 20 and 40 years, history of primary infertility, at least secondary school educational level, and gestational age between 14 to 20 weeks. Exclusion criteria were fetal abnormality, self-reported history of mental illnesses, chronic physical diseases and experience of unfortunate events in the past 3 months (e.g. relative loss).

### Sampling and randomization

Sampling was initiated after obtaining an ethics code from the Ethics Committee of Tabriz University of Medical Sciences (IR.TBZMED.REC.1397.625) and registering the study on the Iranian Registry of Clinical Trials (IRCT20111219008459N12) website. The researcher attended the Al-Zahra Teaching Hospital of Tabriz and extracted the list of women impregnated by assisted reproduction technologies (ART). Then, they were called and provided with a brief description of research objectives and methodology. Eligibility of the samples was assessed and eligible women who were willing to participate in the study were invited. In an in-person meeting, research objectives and methodology were completely explained. Women impregnated with ART, were interviewed and their Perceived Stress Scale (PSS) was completed by the author. The participants scored higher than the cut-off point of 21.8 (high level of stress) were included and their informed written consent was obtained. Then, the Postnatal Depression Scale (PDS), Pregnancy-Related Anxiety Questionnaire (PRAQ), and Quality of Life in Pregnancy (QOL-GRAV) were completed by the researcher through interviews.

The participants were divided into the intervention and control groups using block randomization with blocks of 4 and 6, based on the age range (20–30 and 30–40 years). For allocation concealment, the type of intervention was written on a piece of paper by a person not involved in sampling and data analysis. Papers were then enclosed in specific envelopes numbered sequentially. Each participant received an envelope on arrival.

### Intervention

The intervention group, comprised of 5–7 participants, received six 60- to 90-min in-person CBT sessions in a quiet and friendly room in the Infertility Clinic of Al-Zahra Teaching Hospital and two telephone sessions once a week by the researcher. The CBT sessions was conducted by a graduate student in midwifery counseling under the supervision of a PhD in clinical psychology who designed the intervention procedure. The intervention was supervised by two person who not involved in the study approximately once every two weeks randomly. For encouraging the eligible women to participate in the study, the importance of psychotherapy and its role in improving the outcome of pregnancy were explained during the recruitment stage of study and after confirming their desire to participate in the study and counseling sessions, they were entered to the study. The day before each counseling session, the time of the session was reminded with telephone call and the motivation to participation in the session was strengthened again. In case of non-participation of any person in any meeting, she was invited to another group to hold the same meeting during the same week, therefore, All 28 members of the intervention group completed all counseling sessions. In addition, the counseling was provided in participants’ native language. A brief explanation of the content of the sessions is as follows [21].

- First session: Inducting, determining the number and duration of each session, sequence of sessions and group regulations, identifying problem, introducing CBT, introducing cognitive-behavioral pattern, describing the problem based on the given pattern, determining objectives, and receiving feedback.
- Second session: Conducting mood assessment, describing problem based on the given pattern, impacts of lack of control on stress process, determining objectives, introducing progressive muscle relaxation and practicing it, and allocating homework assignments: planning for progressive muscle relaxation twice per day and receiving feedback.
- Third session: Conducting mood assessment, reviewing progressive muscle relaxation, asking participants to pose problems, introducing imagination and practicing it (Cognitive imagery is a state in which a person visualizes important situations in the mind and mentally anticipates problems and obstacles), and allocating homework assignments: practicing progressive muscle relaxation and imagination, and receiving feedback.
- Fourth session: Conducting mood assessment, reviewing imagination practices, introducing three first columns of thought record sheet (situation, automatic thoughts, emotions, and mood), introducing hot thought concept, practicing three columns (situation, automatic thoughts, emotions, and mood) using one event happened last week, and allocating homework assignments: imagination technique and three first columns of the thought record sheet, receiving feedback.
- Fifth session: Conducting mood assessment, conducting discussion about treatment process and its completion, reviewing homework assignments, introducing cognitive distortions, completing three columns of thought record sheet (situation, automatic thoughts, emotions, and mood), identifying hot thought, introducing the concept of hot thought challenge, and determining homework assignments: relaxation technique and recording three columns of the thought record sheet (situation, automatic thoughts, emotions, and mood) and receiving feedback.
- Sixth session: Conducting mood assessment, reviewing homework assignments, introducing challenge with thoughts and 7-column thought record sheet (situation, automatic thoughts, emotions and mood, confirmatory evidence, rejective evidence, alternative thought, and re-assessment), completing columns during the session, introducing the concept of hot thought challenge, allocating homework assignments: recording three first columns of the though record sheet and relaxation techniques, receiving feedback.
- Seventh session: Conducting mood assessment, reviewing homework assignments, completing seven columns of the thought record sheet during the session (situation, automatic thoughts, emotions and moods, confirmatory evidence, rejective evidence, alternative thought, and re-assessment), and allocating homework assignments: completing 7-column thought record sheet and relaxation techniques, receiving feedback.
- Eighth session: Conducting mood assessment, reviewing homework assignments, reviewing the treatment process (reviewing and recording different CBT techniques), taking recurrence prevention process, introducing self-management sessions and its scheduling, and allocating homework assignments: self-management session and determination of the precise date of post-test.

The control group only received routine pregnancy care based on the “National Guideline for Midwifery and Delivery Services” of Iran [22]. Four weeks after the intervention, the posttest PSS, EPDS, PRAQ, and QOL-GRAV were completed by the author for both groups via a telephone call.

### Data collection tools

Data were collected using the socio-demographic and midwifery scale, Cohen’s perceived stress scale, PRAQ, EPDS, QOL-GRAV.

The socio-demographic and midwifery questionnaire was prepared by the authors and its validity was confirmed by 10 academic members of Tabriz University of Medical Sciences.

#### Cohen’s perceived stress scale

The degree of stress (primary outcome) was measured using PSS-14. The scale is scored based on a 5-point Likert scale; the lowest and highest scores are 0 and 56, respectively. The cut-off point was 21.8 and a higher score indicated a higher degree of stress. The internal consistency and reliability coefficients were obtained through Cronbach’s alpha in a range of 0.84–0.86 in two groups of students and a group of tobacco users in a smoking cessation program [23]. The reliability of the Farsi version was calculated by Bastani et al., using the internal consistency method and the Cronbach’s alpha of 0.74 was obtained [24].

#### Van Den Burgh’s pregnancy-related anxiety questionnaire

The shortened version of the PRAQ (PRAQ-17) which was used to measure the anxiety level (secondary outcome), contains 17 items and the score of each item ranges between 1 and 7. Therefore, the total PRAQ score is between 17 and 119. The higher total PRAQ score indicates a higher level of anxiety [25].

#### The Edinburgh postnatal depression scale

This scale is used to measure the level of depression (secondary outcome) during pregnancy and postpartum. Items are scored from 0 to 3 based on the severity of symptoms. An individual’s score is the total sum of all 10 items, which varies between 0 and 30. Mothers who scored higher than the threshold level [12] had different levels of depression [26]. In the present study, such mothers were referred to a psychologist.

#### Quality of life in pregnancy (Gravidarum) (QOL-GRAV) questionnaire

This questionnaire was developed by Vachkova et al. (2013) using the WHO Quality of Life-BREF (WHOQOL-BREF). It is composed of 9 items to measure individuals’ experience of the quality of life during pregnancy (secondary outcome). The QOL-GRAV’s score varies between 9 and 63. Higher scores indicate a higher quality of life [27]. Mirghafoorvand et al. indicated acceptable validity and reliability of the questionnaire [28].

In the present study, the reliability of the questionnaire was determined through test-retest on 20 women in a two-week interval and the Cronbach’s alpha and intraclass correlation coefficient (ICC) was determined. The ICC for PSS, PRAQ, EPDS, and QOL-GRAV was 0.91, 0.88, 0.83, and 0.93, respectively. The Cronbach’s alpha for these questionnaires was 0.87, 0.78, 0.72, and 0.91, respectively.

Existence of any adverse event after the intervention was asked from participants as an open question after completing the post-test questionnaires.

### Statistical analysis

After collecting data from all participants, they were analyzed in SPSS-21. The normality of the quantitative data was determined using the Kolmogorov-Smirnov Test and all variables were normal. The chi-square test, Trend chi-square test, Fisher’s exact test, and independent t-test were used to assess the consistency of socio-demographic data. The independent t-test was used to compare the two groups before intervention in terms of perceived stress, anxiety, depression, and quality of life. After the completion of the intervention, this comparison was conducted using the analysis of covariance (ANCOVA) while controlling the baseline values and the effect size was calculated based on this formula: Cohen’s *d* = (*M*_2_ - *M*_1_) / *SD*_pooled_. All analyses were done based on intention-to treat.

Sample size estimation in G-Power was 24 per group based on the study by Hassan-Zadeh Lifshagerd [29], considering m1 = 35.2 (mean score of perceived stress), and presumed stress score reduction by 15% after the intervention (m2 = 29.92; sd1 = sd2 = 4.87; two-sided α = 0.05; power = 95%). The final sample size was 28 considering the probable sample loss of about 15%.

## Results

**Fig. 1:**
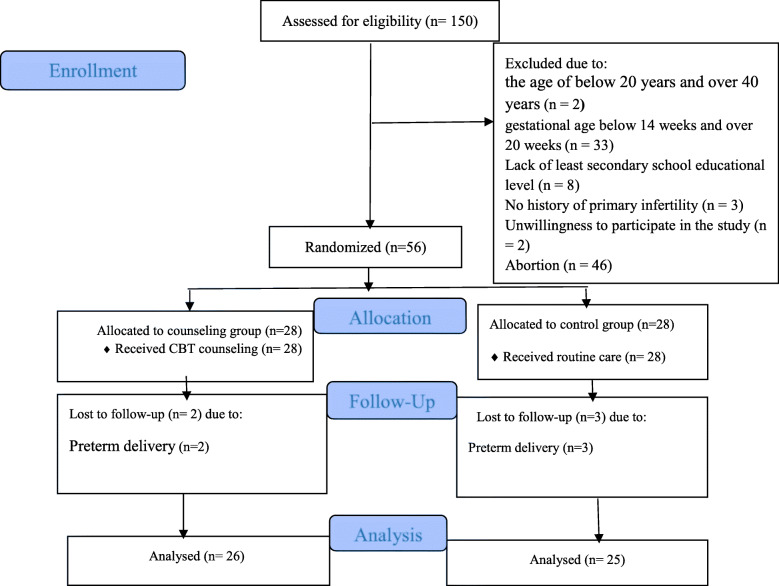
Flow chart of the study

At the beginning of the study, 150 women with positive pregnancy test results were contacted. Finally, 94 women were excluded based on eligibility criteria. The research instruments were completed for the remaining women (*n* = 56) and they were equally randomized into counseling and control groups. Because the participants were motivated enough by the researcher, all 28 participants of the intervention group completed all counseling sessions, they also did their homework in more than 90% of the cases. Due to premature birth, two participants in the counseling group and three participants in the control group were excluded. As a result, the post-intervention follow-up analysis was conducted with 26 participants in the counseling group and 25 participants in the control group (Fig. 1).

The comparison of socio-demographic characteristics of participants in study groups was shown in Table 1. There was no significant between-group difference in socio-demographic data, except gestational age and husband educational attainment (Table 1). However, both variables were moderated using the ANCOVA (*p* > 0.05).

**Table 1:** Socio-demographic characteristics of participants in study groups

Characteristic	Counseling (*n* = 28) number (%)	Control (*n* = 28) number (%)	*P*-value
Age (years) *	31.8 (5.85)	31.1 (5.3)	0.668^†^
Husband’s age (years) *	37.1 (7.2)	36.1 (5.0)	0.551^†^
Duration of infertility (years) *	8.1 (5.2)	7.5 (4.5)	0.663^†^
Gestational age (years) *	15.9 (2.4)	17.1 (2.6)	0.046^†^
Causes of Infertility			0.515^††^
For men	21 (75.0)	23 (82.1)	
Feminine	7 (25.0)	5 (17.9)	
Level of education			0.240^‡^
Secondary school	3 (10.7)	9(32.1)	
High school	4 (14.3)	2(7.1)	
Diploma	11 (39.3)	8(28.6)	
University	10 (35.7)	9(32.1)	
Job			0.143^§^
Housewife	21(75.0)	26(92.9)	
Employed	7(25.0)	2(7.1)	
Husband’s education			0.034^‡^
Illiterate	0(0)	2(7.1)	
Elementary	0(0)	4(14.3)	
Secondary school	3(10.7)	5(17.9)	
High school	4(14.3)	2(7.1)	
Diploma	10(35.7)	6(21.4)	
Academic	11(39.3)	9(32.1)	
Husband’s occupation			0.265^§^
Jobless	1(3.6)	0(0)	
Employee	12(42.9)	7(25.0)	
Worker	5(17.9)	8(28.6)	
Shopkeeper	7(25.0)	12(42.9)	
Other	3(10.7)	1(3.6)	
Monthly income level			0.752^‡^
Adequate	6(21.4)	7(25.0)	
Inadequate	7(25.0)	2(7.1)	
Relatively adequate	15(53.6)	19(67.9)	
House status			0.310^§^
Personal	16(57.1)	19(67.9)	
Rental	6(21.4)	2(7.1)	
Woman’s parents’ house	1(3.6)	0(0)	
Husband’s parents’ house	5(17.9)	7(25.0)	
Life satisfaction			0.440^‡^
Completely	25(89.3)	23(82.1)	
Relatively	2(7.1)	3(10.7)	
Unsatisfied	1(3.6)	2(7.1)	
Having a history of domestic violence	0(0)	0(0)	
Frequency of treatment failure			0.823^‡^
Zero	5(17.9)	4(14.3)	
One	7(25.0)	6(21.4)	
Twice and more	18(64.2)	16(67.)	

*mean(SD)

^§^ Fisher’s exact test

†Independent t-test

^††^ Chi-square test

^‡^ Trend Chi-square test

The independent t-test showed no significant between-group differences of the total perceived stress score before intervention (*p* = 0.561). Based on the ANCOVA and adjusted baseline values, the mean ± standard deviation of the total perceived stress score was significantly lower in the counseling group than that of control group after intervention (mean difference = − 7.3; confidence interval of 95%: from − 0.9 to − 5.6; *p* < 0.001) (Table 2), corresponding to a large effect size (2.48).

**Table 2:** Comparison of mean perceived stress score of anxiety, depression and quality of life in the Study Groups

Variable	Counseling group mean (SD)	Control group mean (SD)	Mean difference (95% confidence interval)	*P*-value
Perceived stress score (score: 0 to 56)				
Before intervention	46.5 (2.6)	46.8 (2.4)	−0.4 (−1.7 to 0.95)	^⁎^561/0
4 weeks after intervention	38.9 (3.9)	46.6 (2.2)	−7.3 (−9.0 to −5.6)	^†^001/0 >
Anxiety Score: (Score: 17 to 119)				
Before intervention	63.2 (11.9)	65.6 (13.8)	−2.4 (−9.3 to 4.5)	^⁎^484/0
4 weeks after intervention	48.0 (9.6)	63.9 (12.4)	−14.7 (−20.6 to −8.8)	^†^001/0 >
Depression Score (score: 0 to 30)				
Before intervention	16.2 (4.7)	17.0 (4.2)	−.82 (−3.2 to 1.6)	^⁎^495/0
4 weeks after intervention	13.1 (3.2)	15.3 (5.3)	−1.95 (−3.9 to 0.2)	^†^052/0
Quality of Life Score (score: 9 to 63)				
Before intervention	25.7 (3.1)	25.5 (4.4)	0.21 (−1.8 to 2.2)	^⁎^833/0
4 weeks after intervention	31.5 (3.1)	25.8 (3.2)	5.4 (3.4 to 7.4)	^†^001/0 >

⁎ Independent t-test

† ANCOVA with baseline score control and gestational age and spouse education variables

Before intervention, the number of participants in the counseling group was 28 and in the control group was 28, and after the intervention in the counseling group was 26 and in the control group was 25

The independent t-test showed no significant between-group differences of total anxiety score before intervention (*p* = 0.484), whereas based on the ANCOVA and adjusted baseline values, the mean ± standard deviation of total anxiety score was significantly lower in the counseling group than that of control group after intervention (mean difference = − 14.7; confidence interval of 95%: from − 20.6 to − 8.8; p < 0.001) (Table 2), corresponding to a large effect size (1.43).

The independent t-test showed no significant between-group differences of total depression score before intervention (*p* = 0.495). After intervention, based on the ANCOVA and adjusted baseline values, the mean ± standard deviation of depression was lower in the counseling group than that of control group; however, this between-group difference was not significant (mean difference = − 1.95; confidence interval of 95%: from 3.9 to − 0.2; *p* = 0.052) (Table 2), corresponding to a medium effect size (0.51).

The independent t-test showed no significant between-group differences of total quality of life score before intervention (*p* = 0.833). After intervention based on the ANCOVA and adjusted baseline values, the mean ± standard deviation of the total quality of life score was significantly higher in the counseling group than that of control group (mean difference = 5.4; confidence interval of 95%: from 3.4 to 7.4; *p* < 0.001) (Table 2), corresponding to a large effect size (1.8).

No adverse events were reported by participants.

## Discussion

Results from this study showed that the mean perceived stress and anxiety scores were significantly lower in the counseling group than the control 4 weeks after intervention. The mean depression score was lower in the control group 4 weeks after intervention with adjusted baseline values; however, this between-group difference was not significant. Moreover, the mean quality of life score was significantly higher in the counseling group than the control.

In a review study, Ying et al. (2016) investigated the effect of psychological interventions on mental health, pregnancy rate, and marital function of infertile couples undergoing IVF. In 20 trials under investigation, 14 interventions, such as CBT, mindfulness, counseling, coping with stress, and positive reassessment used at different IVF stages were studied. The authors concluded that none of these interventions were effective in soothing stress and depression of patients undergoing IVF [30]. Results from the above study were inconsistent with the present study, which could be attributed to the following reasons. The present study investigated the use of CBT on pregnant women with history of primary infertility; whereas, Ying et al. implemented interventions during the course of treatment. Moreover, none of the reviews investigated the psychological outcomes of intervention in the two-week wait period, despite the fact that this period is one of the most difficult times in the life of infertile couples.

Given that stress perception and responding to it are affected by previous experiences, the present situation and learned behaviors [31], it could be concluded that the CBT counseling in which new thinking and behavior techniques are taught to replace negative thoughts of patients about self, world, and future [32, 33], are helpful in identifying stressful situations and using coping strategies. The correction of cognitive assessments, improvement of coping skills, and combination of practices to integrate learned techniques with real-life situations can reduce the level of stress [29].

Hamzeh Poor (2014) showed that anxiety in infertile women was significantly lower in the CBT group than the control [34]. Findings of the present study were consistent with those of the above study. However, Hamzeh Poor investigated the participants from the initial treatment to the intrauterine insemination (IUI) stage, i.e. before getting positive pregnancy result. In a quasi-experimental study, Salehi (2016) compared the effectiveness of group CBT and interactive lecturing (IL) in reducing anxiety in pregnancy. Results showed a significant reduction in the state and trait anxiety in CBT and IL groups after 4 weeks (*p* < 0.001). In addition, group CBT was more effective than interactive lectures in reducing participants anxiety; however, this between-group difference was not significant (*p* > 0.05) [35]. Results from the above study were consistent with the findings of the present study; however, there was a difference between two studies in the number of counseling sessions and participants.

In a review and meta-analysis study, Golshani et al. (2020) examined the effect of cognitive-behavioral therapy on anxiety and depression in Iranian infertile women and concluded that the level of anxiety in infertile women who received cognitive-behavioral therapy compared to the control group was significantly lower (*P* < 0.001); But it had no significant effect on depression [36]. The results of the above study are consistent with the present study. Abdolahi et al. (2019) in a meta-analysis study examined the effect of cognitive-behavioral therapy on anxiety and depression in infertile women infertile women with or without IVF / ICSI and concluded that cognitive-behavioral therapy leads to a significant reduction in anxiety (P < 0.001) and Depression (P < 0.001) in the counseling group compared to the control group [37]. The results of the above study are consistent with our study in terms of the effect of counseling on anxiety, but do not agree with our study on depression. The reason for this difference can be attributed to the different samples. Imanparast et al. (2014) showed that CBT can significantly reduce anxiety in nulliparous women [38]. Chatwin et al., (2016) investigated the effectiveness of CBT and emotional freedom technique (EFT) in reducing depression and anxiety in adults and showed that both methods significantly reduced depression symptoms in adults with major depression. However, the post-intervention anxiety score was not significantly diffident from the pretest score (*p* = 0.104). Moreover, anxiety score in the CBT group was significantly lower than that of EFT group (*p* = 0.032) [39].

According to the cognitive-behavioral theory, anxiety disorders are caused by mistaken beliefs, which affect the interpretation of events and induce a disproportionate emotional response [40]. As a result, holding counseling sessions for muscle relaxation, and identification of challenging thoughts and beliefs can replace the wrong attitudes of pregnant women with rational ones, indicating the effectiveness of CBT in anxiety management [28, 41]. Studies have shown that infertility treatment failure may cause permanent emotional burden in infertile women [42, 43]. To explain the results, in many participants, negative experiences, such as infertility treatment costs, continuous worries about treatment outcomes, fatigue from frequent visits to medical centers, curiosity of relatives, fear of family breakdown, and fear of losing husband interest before and during mental and social stress assessment resulted in a sense of helplessness, conflict, frustration, sharp decline in self-esteem and self-confidence, and isolation [44]. These severe mental stressors play a significant role in depression. As a result, our intervention may be insufficient for addressing many of the participants’ psychosocial needs.

In this study, cognitive-behavior counseling improved the quality of life of pregnant women with a history of primary infertility. In the review of literature, the author found no relevant study on pregnant women. In a controlled randomized clinical trial, Cooney et al. (2018) investigated the effect of CBT on weight and quality of life of women with polycystic ovary syndrome (PCOS). Results showed that weekly CBT + LS (lifestyle) for 8 weeks was more effective than LS alone in reducing weight and improving the quality of life in women with PCOS [45]. In a quasi-experimental study, ‘Isa-Zadegan et al. (2013) showed that CBT in patients with hypertension can significantly increase the mean quality of life score in the counseling and control groups (*p* < 0.01) [46]. In a clinical trial, Jalilian et al. (2018) showed that the CBT can affect and enhance the psychological and physical components of the quality of life in women with PCOS (*p* < 0.05) [47]. Results from the above study were consistent with the present study; however, there were between-study differences in the target population, CBT type, and the number of follow-up sessions.

All participants in this study were first-time pregnant women with a history of primary infertility and were literate**,** while the most of previous studies were either about CBT-based counseling in pregnant women with no history of infertility or were about the impact of this type of counseling on the mental health of infertile women during infertility treatment before getting pregnancy test results and in some studies the target population was quite different, which should be considered in comparing the results.

The financial burden from infertility treatment, lengthy treatment period, irrational thoughts about having a child, psychological pressures from relatives, and low educational level are among factors having adverse effects on the quality of life of infertile women [48]. Women impregnated after these difficult stages experience a very stressful and challenging pregnancy [49]. The researchers believe that although infertility, as a source of psychological pressure, can endanger mental health of infertile people, its effect depends on the psychological assessment and coping skills of those people. Therefore, teaching these skills to control emotions plays a significant role in reducing psychological pressures caused by infertility-induced stress [6]. It could be concluded that CBT can cause some changes in the psychological dimensions of pregnant women. When these women use CBT skills in stressful situations, they feel capable of making decisions, controlling their life events, and taking effective measures to achieve desirable results. They internally feel satisfaction which, in turn, increases happiness, mental well-being, and self-efficiency, as the quality of life factors [46].

In addition to the positive effects mentioned for CBT, because this method is done in a group, it also has the benefits of group therapy. In this way, people see themselves as part of a group that has had similar experiences; As a result, they know that what happened to them is common and they are not alone. Group members help each other by exchanging information. Sharing feelings and experiences with a group of people helps to relieve feelings of pain, guilt or stress and causes emotional relief.

### Limitations and strengths

In the present study, all responses of participants were assumed to be correct as their validation was beyond the researchers’ ability. Moreover, this study was a small study in one center and all participants were literate with history of primary infertility, which could affect generalizability of the results, therefore, the results should be generalized with caution. Among the strengths of this study are observing all principles of clinical trials, including allocation randomization and allocation concealment, completion of the questionnaire by the researcher, and reduction of plausible incomplete, null, and wrong responses. To make a better communication with the participants, their native language was used during the counseling sessions.

## Conclusion

According to the results, CBT-based group counseling is effective in reducing perceived stress and anxiety, and improving quality of life. Given the needs of pregnant women with a history of primary infertility for both psychical and psychological supports to improve pregnancy outcomes, mental health, and quality of life, healthcare providers can provide them with this counseling technique, along with routine pregnancy care.

## Data Availability

Data and materials of this study are available from the corresponding author upon reasonable request.

## Abbreviations

CBT: Cognitive Behavioral Therapy
QoL: Quality of Life
PSS: Perceived Stress Scale
EPDS: Edinburgh Postnatal Depression Scale
PRAQ: Pregnancy-Related Anxiety Questionnaire
QoL-GRAV: Quality of Life in Pregnancy (Gravidarum)
